# Retraction: IoT based battery energy monitoring and management for electric vehicles with improved converter efficiency

**DOI:** 10.1371/journal.pone.0334668

**Published:** 2025-10-16

**Authors:** The PLOS One Editors

The *PLOS One* Editors retract this article [1] due to concerns about authorship and adherence to the journal’s publication requirements on reporting and data availability. During editorial follow-up, the authors provided additional information and code which were insufficient to resolve the concerns. We regret that the issues were not addressed prior to the article’s publication.

All authors did not agree with the retraction.
